# Sexually dimorphic patterns in maternal circulating microRNAs in pregnancies complicated by fetal growth restriction

**DOI:** 10.1186/s13293-021-00405-z

**Published:** 2021-11-17

**Authors:** Bernadette C. Baker, Sylvia Lui, Isabel Lorne, Alexander E. P. Heazell, Karen Forbes, Rebecca L. Jones

**Affiliations:** 1 Division of Developmental Biology and Medicine, Maternal and Fetal Health Research Centre, University of Manchester, Manchester, UK; 2 Division of Inflammation and Repair, School of Medical Sciences, Faculty of Biology, Medicine and Health, University of Manchester, Manchester, UK; 3 St Mary’s Hospital, Manchester University NHS Foundation Trust, Manchester Academic Health Science Centre, Manchester, M13 9WL UK; 4 Discovery and Translational Science Department, Leeds Institute of Cardiovascular and Metabolic Medicine, University of Leeds, Leeds, UK

**Keywords:** miRNA, Placenta, Pregnancy, Serum, Biomarker, Placental dysfunction, FGR, Stillbirth, Sexual dimorphism

## Abstract

**Background:**

Current methods fail to accurately predict women at greatest risk of developing fetal growth restriction (FGR) or related adverse outcomes, including stillbirth. Sexual dimorphism in these adverse pregnancy outcomes is well documented as are sex-specific differences in gene and protein expression in the placenta. Circulating maternal serum microRNAs (miRNAs) offer potential as biomarkers that may also be informative of underlying pathology. We hypothesised that FGR would be associated with an altered miRNA profile and would differ depending on fetal sex.

**Methods:**

miRNA expression profiles were assessed in maternal serum (> 36 weeks’ gestation) from women delivering a severely FGR infant (defined as an individualised birthweight centile (IBC) < 3rd) and matched control participants (AGA; IBC = 20–80th), using miRNA arrays. qPCR was performed using specific miRNA primers in an expanded cohort of patients with IBC < 5th (*n* = 15 males, *n* = 16 females/group). Maternal serum human placental lactogen (hPL) was used as a proxy to determine if serum miRNAs were related to placental dysfunction. In silico analyses were performed to predict the potential functions of altered miRNAs.

**Results:**

Initial analyses revealed 11 miRNAs were altered in maternal serum from FGR pregnancies. In silico analyses revealed all 11 altered miRNAs were located in a network of genes that regulate placental function. Subsequent analysis demonstrated four miRNAs showed sexually dimorphic patterns. miR-28-5p was reduced in FGR pregnancies (*p* < 0.01) only when there was a female offspring and miR-301a-3p was only reduced in FGR pregnancies with a male fetus (*p* < 0.05). miR-454-3p was decreased in FGR pregnancies (*p* < 0.05) regardless of fetal sex but was only positively correlated to hPL when the fetus was female. Conversely, miR-29c-3p was correlated to maternal hPL only when the fetus was male. Target genes for sexually dimorphic miRNAs reveal potential functional roles in the placenta including angiogenesis, placental growth, nutrient transport and apoptosis.

**Conclusions:**

These studies have identified sexually dimorphic patterns for miRNAs in maternal serum in FGR. These miRNAs may have potential as non-invasive biomarkers for FGR and associated placental dysfunction. Further studies to determine if these miRNAs have potential functional roles in the placenta may provide greater understanding of the pathogenesis of placental dysfunction and the differing susceptibility of male and female fetuses to adverse in utero conditions.

**Supplementary Information:**

The online version contains supplementary material available at 10.1186/s13293-021-00405-z.

## Highlights

- Detection and treatment of pregnancies at high risk of fetal growth restriction (FGR) and stillbirth remains a major obstetric challenge; circulating maternal serum microRNAs (miRNAs) offer potential as novel biomarkers.
- Unbiased analysis of serum miRNAs in women in late pregnancy identified a specific profile of circulating miRNAs in women with a growth-restricted infant.
- Some altered miRNAs (miR-28-5p, miR-301a-3p) showed sexually dimorphic expression in FGR pregnancies and others a fetal-sex dependent association to a hormonal marker of placental dysfunction (miR-454-3p, miR-29c-3p).
- miR-301a-3p and miR-28-5p could potentially be used to predict FGR specifically in pregnancies with a male or female baby, respectively, however larger cohort studies are required.
- Further investigations of these miRNAs and their relationship to placental dysfunction will lead to a better understanding of the pathophysiology of FGR and why there is differing susceptibility of male and female fetuses to FGR and stillbirth.

**Supplementary Information:**

The online version contains supplementary material available at 10.1186/s13293-021-00405-z.

## Introduction

Fetal growth restriction (FGR) is a pregnancy complication where the fetus fails to reach its genetically determined growth potential and is a significant cause of fetal morbidity and mortality [1]. FGR is associated with a range of placental abnormalities, including impaired growth, villous and vascular structural abnormalities, and impaired nutrient transport and endocrine function [2, 3]. Fetal growth restriction and associated placental dysfunction are linked to increased rates of stillbirth, death of a fetus in utero after 24 weeks gestation [4]. Stillbirth affects 1 in 240 pregnancies in the UK [5], thus identifying pregnancies with placental dysfunction at greatest risk of FGR and stillbirth is an important clinical aim [2].

The only current direct test of placental function in utero in routine clinical use for detection of FGR is Doppler ultrasound assessment of umbilical artery blood flow [2] however, although this screen is sensitive for detecting early-onset FGR (< 34 weeks’ gestation), it is not reliable for late-onset FGR which comprises the majority of all FGR cases [6]. Whilst other tests in late pregnancy such as measurement of placental hormones in maternal blood, e.g. placental growth factor (PlGF), human placental lactogen (hPL) have also been proposed to identify placental dysfunction, a recent systematic review and meta-analysis demonstrated such tests are insufficient to predict FGR pregnancies thus additional biomarkers are required [7].

MicroRNAs are short (~ 20 nucleotide) RNA molecules that post-transcriptionally regulate gene expression by mediating mRNA destabilisation and translational repression [8]. miRNAs are produced in all cells and tissues, including the placenta, where they regulate growth, differentiation, survival and vascular development [9–12]. Vascular maldevelopment and altered trophoblast turnover and function are features of placental dysfunction observed in FGR and pregnancies ending in stillbirth [13] suggesting that the underlying pathology may be a consequence of altered miRNA levels in the placenta. miRNAs have also been shown to be released from tissues, including the placenta, into the circulation either in complexes with proteins such as Argonaute 2 or contained within extracellular vesicles (EVs) [14–16]. In the maternal circulation, specific alterations to miRNA signatures are associated with pregnancy pathologies such as pre-eclampsia (PE), gestational diabetes and pre-term labour [17–20]. Furthermore, in pregnant mice, plasma miRNAs have been proposed to offer a non-hormonal biomarker for direct indication of placental dysfunction [21], suggesting that circulating miRNAs may also offer promise as biomarkers to predict FGR and associated placental dysfunction in human pregnancy.

Indeed, changes in circulating miRNAs in pregnancies complicated by FGR have been reported [22–25], however, none of the miRNAs currently identified have sufficient diagnostic accuracy for use as biomarkers for FGR. This is likely due to key differences between study design, for example the analysis of a limited number of preselected candidate miRNAs rather than global profiling of miRNAs [22, 26]; differences in gestational ages of when serum was drawn between studies [24, 25]; analysing miRNA content in isolated EVs in some studies versus total serum in others [24]; and analysing obstetric populations with mixed FGR subtypes including women with PE and FGR [23]. To date, no published studies have stratified for fetal sex, a variable known to affect both rates of FGR, stillbirth and placental miRNA expression [27–30]. Therefore, we constructed our study to perform unbiased assessment of miRNAs, instead of selecting for specific candidate miRNAs; to profile total serum rather than EV enriched serum; to use a well-defined FGR population with no other known obstetric/medical complications; to stratify for fetal sex; to use in silico analyses to identify miRNAs involved in placental regulatory networks; and to combine analysis of miRNA expression profiles with levels of a known marker of placental dysfunction. We hypothesised that using this methodology, we would identify miRNA profiles that were associated with both FGR and placental dysfunction and that these miRNA profiles may be dependent on the presence of a male or female fetus.

## Methods

### Participants and clinical samples

Study participants were pregnant women receiving antenatal care at St Mary’s Hospital, Manchester, recruited to the MFHRG Biobank (ethical approval: North West REC (08/H1010/55 + 5)). Written informed consent was obtained and detailed demographic and biophysical data were recorded. Exclusion criteria were multiple pregnancy, pre-term deliveries (< 36 weeks gestation), known fetal anomalies, pre-eclampsia or hypertension, maternal diabetes or any other co-existing obstetric/medical complications. Maternal serum samples were collected in the third trimester (27–42 weeks’ gestation). Briefly maternal venous blood drawn into serum gel tubes (Sarstedt, Numbrecht, Germany) was allowed to clot for 30 min and centrifuged at 3000 *g* for 10 min. Aliquots were immediately frozen at − 80 °C for analysis. After delivery, obstetric outcome data were collected and the individualised birthweight centile (IBC) calculated using Gestation Related Optimal Weight (GROW) Centile Calculator version 6.7 [31]. Babies were considered FGR when IBC < 5th centile. All FGR samples were matched on a 1:1 basis to appropriately grown (AGA; IBC ≥ 20 ≤ 80th centile) fetuses for the following maternal demographic and biophysical characteristics: infant sex (50:50 female to male), gestation at time of blood sampling, and gestation at delivery. Initial miRNA array profiling was performed on a subset of maternal serum samples drawn between 36–37 weeks’ gestation where primary selector was delivery of a baby with severe FGR (IBC < 3rd centile) (*n* = 4/group; Table 1). For all other analyses, a larger cohort of 62 participants’ samples (male *n* = 15, female *n* = 16; per group; Table 2), were included and FGR was defined as IBC < 5th centile. In both array samples and the larger cohort, only infant birthweight and IBC were significantly different by design between groups (array samples *p* < 0.05, larger cohort *p* < 0.0001, Mann–Whitney; Tables 1 and 2).

**Table 1:** Demographic and obstetric outcome data for groups used for microRNA array profiling

Category	AGA *n* = 4	FGR (IBC < 3rd) *n* = 4
Age (years)	28 (27–35)	30.8 (27–35)
Ethnicity Caucasian:other Number (%)	2:2 (50%:50%)	2:2 (50%:50%)
Non-smoker Number (%)	4 (100%)	4 (100%)
BMI at booking kg/m^2^	24.45 (23–28)	24.05 (22.3–28.9)
Gestation at delivery (days)	280 (272–284)	278.5 (264–284)
Birthweight (gramme)	3207 (3040–3560)	2225* (1880–2880)
IBC	32.8 (21.5–46.8)	0.95* (0.1–3.0)
Male infant Number (%)	2 (50%)	2 (50%)
Gestation of blood draw (days)	252 (252–252)	252 (252–262)

Data are median and range unless stated otherwise. Participants were matched 1:1 on all demographic and obstetric factors, except for birthweight and individualised birthweight centile which were significantly different between groups (*p < 0.05 FGR vs AGA, Mann–Whitney test). Data analysed by Mann–Whitney (continuous data) or Fisher’s exact test (categorical data). *AGA* appropriate for gestational age, *BMI* body mass index, *FGR* fetal growth restriction, *IBC* individualised birthweight centile

**Table 2:** Demographic and obstetric outcome data for study participants used for miRNA QPCR analyses stratified by infant sex

Category	AGA male (*n* = 15)	FGR male (*n* = 15)	AGA female (*n* = 16)	FGR female (*n* = 16)
Age (years)	24 (18–40)	28 (18–40)	29 (22–39)	27.5 (20–36)
Ethnicity Caucasian:other Number (%)	10:5 (67%:33%)	8:7 (53%:47%)	7:9 (44%:56%)	10:6 (63%:37%)
Smoker Number (%)	4 (27%)	4 (27%)	1 (6%)	1 (6%)
BMI at booking kg/m^2^	25.6 (21.8–29.8)	24.9 (18.7–30.9)	23.8 (19.0–28.6)	25.0 (19.4–30.5)
Gestation at delivery (days)	282 (257–297)	280 (260–286)	280 (265–293)	274 (256–293)
Birthweight (g)	3560 (3094–4640)	2520**** ^a^ (1960–3190)	3390 (2674–3856)	2370***^b^ (1660–2880)
IBC	41 (20.7–81.3)	1.54**** ^a^ (0.002–5)	54.1 (20.7–81.1)	0.69**** ^b^ (0.1–3.5)
Gestation of blood draw (days)	252 (192–287)	252 (199–281)	252 (197–291)	257 (200–282)

Data are median and range unless stated otherwise. Data analysed by Kruskal–Wallis with Dunn’s post hoc test (continuous data) or Fisher’s exact test (categorical data). *AGA* appropriate for gestational age, *BMI* body mass index, *FGR* fetal growth restriction, *IBC* individualised birthweight centile. ***p < 0.001, ****p < 0.0001; ^a^FGR male compared to AGA male; ^b^FGR female compared to AGA female; (Kruskal–Wallis with Dunn’s post hoc test)

### RNA isolation

Total RNA was extracted from 200 µl of maternal serum using the miRCURY RNA Isolation Kit—Biofluid (Exiqon, Denmark) following the manufacturers’ protocol, with inclusion of an on column DNAse digestion step. For increased reproducibility, a carrier RNA (1 µg MS2 phage RNA; Roche, UK)) was added prior to extraction along with RNA spike-in controls (UniSp2, UniSp4, UniSp5 Exiqon, cat no. 203203) to monitor the technical quality of the RNA isolation, cDNA synthesis and the presence of PCR inhibitors. Samples contaminated by haemolysis where ΔCt miR-23a-3p–miR-451a was > 7 were excluded. Standard methods of RNA yield/purity are inaccurate due to carrier RNA, however we adhered to best practise guidelines by standardising input amounts based on isolating from identical starting volumes and using the same volume of purified RNA for all downstream processes [32].

### miRNA array profiling

Isolated maternal serum RNA (19 µl) was reverse transcribed in 95-µl reactions using the miRCURY LNA™ Universal RT microRNA PCR system (Exiqon, Denmark) then diluted 50 × before analysis using the miRCURY LNA™ Universal RT microRNA PCR Human panel I + II Array plates (Exiqon, Denmark) which detects 752 individual miRNAs (list of all miRNAs profiled contained in Additional file 1). Sample quality control was monitored using spike-ins for RNA isolation efficiency (UniSp2, UniSp4, UniSp5) and cDNA synthesis control (UniSp6). Individual miRNA Ct values in each sample, were normalised to the global mean of a panel of 194 miRNA Ct values detected in the same sample (the 194 miRNAs were detected in all samples) as recommended by Mestdagh et al. [33]. The stability of the average of the 194 microRNAs was higher across samples/groups than any single miRNA in the dataset as measured by NormFinder software [34]. The coefficient of variation (CoV) for the array was 0.00024–0.048%. Detection of statistical differences between patient groups was performed using a paired *t*-test followed by Benjamini and Hochberg multiple testing correction, using the software R/Bioconductor [35]. miRNAs with fold-change increase or decrease of more than 1.2-fold (≤ 0.83 ≥ 1.2) change *p* < 0.05 were considered significant.

### Bioinformatic analysis of altered miRNAs

A list of all 11 miRNAs altered in maternal serum from FGR pregnancies was uploaded to Ingenuity Knowledge Base (Ingenuity Pathway Analysis (IPA), Qiagen, Redwood City, www.qiagen.com/ingenuity) and miRNET version 2.0 [36], a freely available tool that can be accessed at https://www.mirnet.ca., which integrates data from 15 different miRNA databases (including miRbase, TarBase, miRTarBase, miRecords, miRanda, TransmiR2.0 miR2Disease, HMDD, ENCODE and ExoCarta) [36], to allow network-based visualisation of miRNA–target gene interactions coupled with functional analysis.

Networks of interacting genes associated with altered miRNAs were determined using miRNET and functional annotation and enrichment analysis of predicted target genes of altered miRNAs was performed using both miRNET (using Reactome, Gene Ontology (GO) and Kyoto Encyclopedia of Genes and Genomes (KEGG) analyses) and IPA. Network connectivity and functions with *p* < 0.05 were regarded as statistically significant.

### Real-time QPCR assessment of serum miRNA levels

Altered miRNAs that had known sexually dimorphic expression in other tissues and/or previous associations with pregnancy pathologies as determined by performing literature searches using PubMed [37] (Table 3), were assessed by QPCR in a larger set of maternal serum samples (Table 2). Reverse transcription of isolated serum RNA (4 µl) was performed using miRCURY™ LNA Universal RT microRNA PCR system (Exiqon, Vedbaek, Denmark), with a UniSp6 RNA spike-in template included as an internal QC to monitor the reaction efficiency. QPCR was performed using ExiLENT SYBR Green master mix (Exiqon) and specific LNA-primer sets for 7 selected candidate miRNAs (miR-28-5p, miR-29c-3p, miR-301a-3p, miR-378a-3p, miR-409-3p, miR-454-3p and miR-526b-5p with target sequences listed in Additional file 2: Table S1) and reference dye 5-carboxy-X-rhodamine (ROX) in a Stratagene MX3000P real-time PCR machine. Thermocycling conditions consisted of polymerase activation for 10 min at 95 °C followed by 40 cycles each one consisting of 10 s at 95 °C then 1 min at 60 °C. The CoV for qPCR assays ranged from 0.2–2.4%. Data were normalised as 2^−ΔCT^ using the geometric mean of two reference miRNAs (miR-23a-3p and miR-191-5p) determined to be most stably expressed between the sample groups as described by Vandesompele et al. [38].

**Table 3:** MicroRNAs differentially expressed in maternal serum between FGR and uncomplicated pregnancies identified by microarray

miRNA ID	SD	ddCq adverse/normal	Fold-change	Unadjusted *p* value	Sexual dimorphism	Reported association with pregnancy pathology
miR-28-5p MIMAT0000085	0.641	− 1.258	− 2.4	0.029	↑ F non-pregnant plasma [39]	Not reported
miR-200b-3p MIMAT0000318	0.169	− 0.874	− 1.8	0.012	↑ M cerebellum + colorectal mucosa [40]	↑ Placental expression in PE with SGA [41] ↑ Myometrial expression in mouse model of PTB [42]
miR-224-5p MIMAT0000281	0.426	− 0.931	− 1.9	0.022	Not reported	↑ Placental expression and maternal serum levels in PE [43] ↑ In trophoblasts exposed to hypoxia [44]
miR-378a-3p MIMAT0000732	0.275	− 0.491	− 1.4	0.038	↑ F normal-term placenta [45]	↑ Placental expression in PTB [46] ↓ Expression in EPL decidua [47]
miR-526b-5p MIMAT0002835	0.420	− 1.463	− 2.8	0.026	↑ M cerebellum [40]	Placental specific C19MC cluster ↓ Placental expression in FGR [48, 49]; ↑ placental expression in PE [50] and PTB [46]
miR-550a-3p MIMAT0003257	0.254	− 0.429	− 1.3	0.043	Not reported	Not reported
miR-29c-3p MIMAT0000681	0.089	0.332	+ 1.3	0.0051	↑ M plasma [39] ↑ F cerebellum [40]	↑ Plasma levels in gestationally obese women [51]
miR-301a-3p MIMAT0000688	0.364	0.588	+ 1.5	0.048	↑ M peripheral blood [40]	↑ Maternal serum levels in PE [43]
miR-409-3p MIMAT0001639	0.422	1.716	+ 3.3	0.02	Plasma concentration + vely correlates with lung function in asthmatic boys [52]	High placental expression (C14MC cluster) ↓ Maternal serum levels in PE [53]
miR-454-3p MIMAT0003885	0.727	1.396	+ 2.6	0.031	↑ M peripheral blood [40] ↑ M FGR fetal heart baboon [54]	↑ Placental expression in PE [55]
miR-551a MIMAT0003214	0.303	0.988	+ 2.0	0.03	Not reported	Not reported

Fold-change in maternal serum expression for FGR < 3rd IBC compared to AGA matched controls profiled by microarray with unadjusted *p* value. Evidence of sexual dimorphism and/or association with pregnancy pathologies was determined by PubMed literature search accessed 30 May 2021. *M* male, *F* female, *PE* pre-eclampsia, *SGA* small for gestational age, *PTB* pre-term birth, *EPL* early pregnancy loss, *FGR* fetal growth restriction. ddCq = (dCq _miRNA_ FGR) – (dCq _miRNA_ AGA) as defined in Livak et al. [56]

### Measurement of maternal serum human placental lactogen (hPL)

Levels of human placental lactogen (hPL), an established marker of placental endocrine function associated with a poor pregnancy outcome [57, 58], were assessed in matched serum samples which had been analysed for miRNA levels, where available (*n* = 59 from a total of 62 samples). Maternal serum samples were diluted 1:100 and assessed using a specific hPL ELISA (EIA-1283, DRG Diagnostics, Germany) according to manufacturer’s instructions. Briefly, 10-µl aliquots of samples and standards were applied to ELISA plates in duplicate and absorbance read at 450 nm using a Versamax plate reader and SoftMax Pro software (Molecular Devices, California, USA). The inter- and intra-assay variabilities were 4.3–9.9% and 2.6–5.5%, respectively. To identify altered miRNAs that were associated with placental dysfunction in FGR, maternal hPL concentrations and 2^−ΔCT^ values for altered circulating miRNAs were plotted for each individual patient sample.

### In silico analysis to identify validated targets of altered miRNAs

Target genes and previously reported associations between altered miRNAs and pregnancy complications associated with placental dysfunction, were identified using in silico analyses. Briefly, miRNA target genes were selected based on previous experimental validation or that were predicted target genes in more than 1 target prediction program (miRbase v.22 [59] miRTarBase Release 8.0 [60], TargetScan Release 7 [61] and miRDB [62]), based solely on the miRNA seed sequence. To further refine the list a literature search was performed using PubMed [37] to identify published studies supporting a functional role of the gene in the placenta or showing associations between gene target expression and pregnancy pathologies associated with placental dysfunction, including pre-term birth (PTB), FGR, PE, early pregnancy loss (EPL) or gestational diabetes (GDM).

### Statistical analysis

Statistical analyses were performed using GraphPad Prism (version 8.4). Demographic and obstetric outcome data were analysed using Mann–Whitney *U* test for continuous data or Fisher’s exact test for categorical data. Data from QPCR analyses were analysed using Mann–Whitney *U* test for 2 groups or 2-way ANOVA for multiple groups. Correlations between miRNAs and hPL were analysed using Spearman’s correlation or linear regression. Results were considered significant if *p* < 0.05. Sample sizes were dictated by power calculations based on previous studies [63] or in the case of validation studies, were determined using a power calculation at 0.95 level of confidence and a *p* < 0.05 based on the microarray values.

## Results

### miRNA profiling of maternal serum samples in FGR and uncomplicated pregnancies

**Fig. 1:**
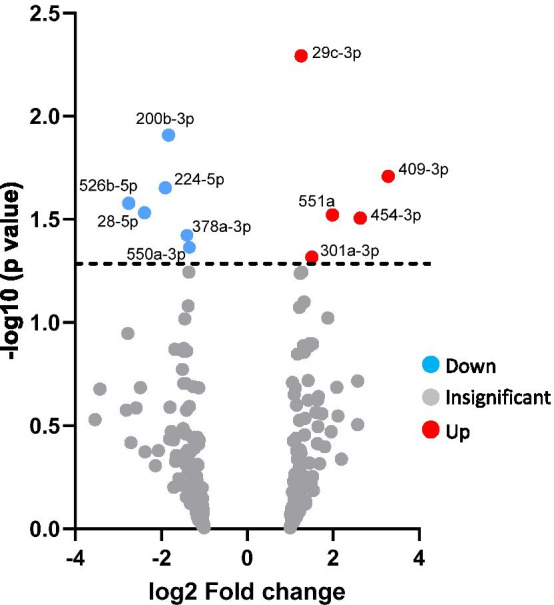
Volcano plot of differentially expressed microRNAs in maternal serum of FGR versus normal pregnancies. Log2 (fold-change) values for detected miRNAs were plotted against − log10 of the unadjusted *p*-value. Significantly up-regulated genes shown in red, significantly down-regulated genes shown in blue (*p* < 0.05). Dotted line represents *p* = 0.05

Analysis of microarrays revealed 11 miRNAs that were altered between maternal serum samples from FGR (IBC < 3rd) and uncomplicated pregnancies at 36 weeks’ gestation (fold-change of ≤ 0.83 ≥ 1.2; *p* < 0.05; Fig. 1). Of these, five miRNAs: miR-29c-3p, 409-3p, 551a, 454-3p and 301a-3p, were increased in FGR and six miRNAs: miR-200b-3p, 224-5p, 526b-5p, 28-5p, 378a-3p and 550a-3p, had reduced levels in FGR serum when compared to AGA controls. Eight of the altered miRNAs: miR-28-5p, 200b-3p, 378a-3p, 29c-3p, 526b-5p, 301a-3p, 409-3p and 454-3p, had previously reported sex differences in expression and /or known association with pregnancy pathologies (Table 3).

### Functional enrichment analysis of altered miRNAs

**Fig. 2:**
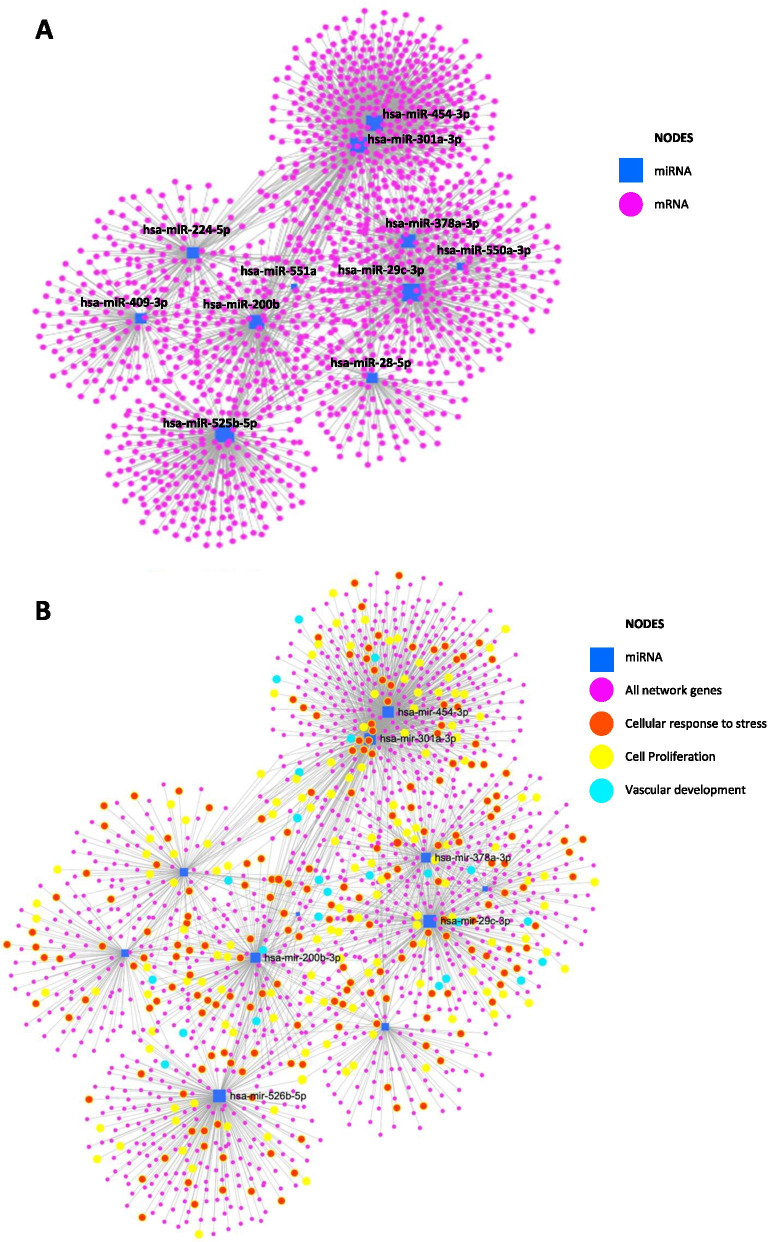
Interacting mRNA networks of all altered miRNAs. **A**, **B** A list of all 11 miRNAs altered in FGR was uploaded to miRNET and **A** networks of interacting genes (pink circles) associated with altered miRNAs (blue square) were determined. **B** Functional enrichment analysis of network genes was performed using Reactome, GO and KEGG. Key functional effects associated with the network were found in pathways associated with cellular response to stress (red; 77 node genes; Adj *p* = 4.9 × 10^–6^), cell proliferation (yellow; 111 node genes; Adj *p* value = 0.00012) and vascular development (turquoise; 97 node genes; Adj *p* value = 0.00037)

**Fig. 3:**
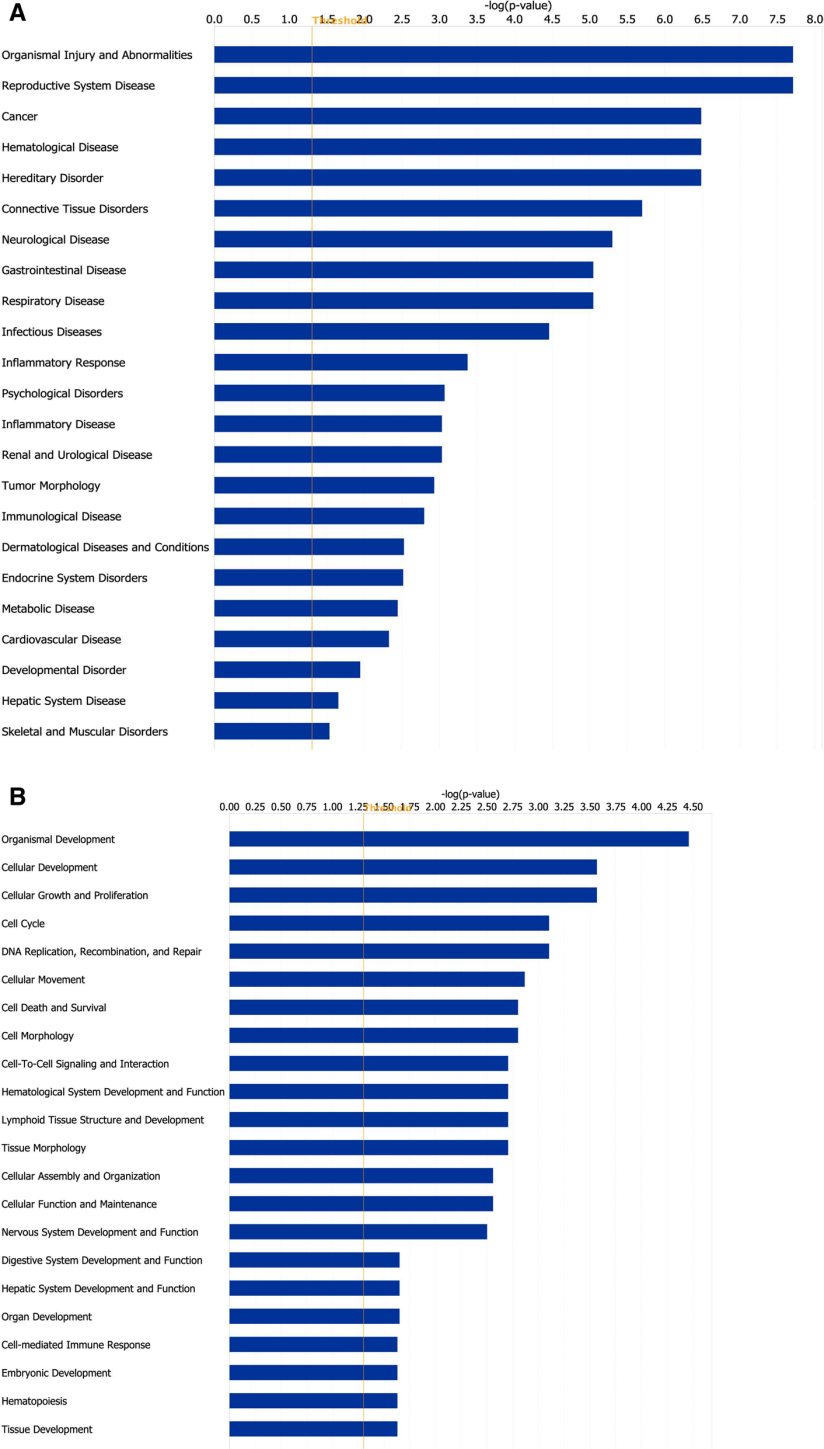
Functional enrichment analysis of miRNAs and predicted target genes. A list of the 11 miRNAs altered in FGR was uploaded to Ingenuity Pathway Analysis and **A** predicted diseases and disorders and **B** predicted molecular and cellular functions and functions associated with physiological system development were determined from miRNAs and their experimentally validated and predicted targets. Orange line represents significance threshold value of − log *p* value = 1.5

Network analysis, using miRNET, revealed that all 11 miRNAs were present in an interacting gene network (Fig. 2A) enriched in predicted target genes (Fig. 2B) associated with cellular response to stress (77 node genes; Adj *p* value = 4.9 × 10^–6^), cell proliferation (111 node genes; Adj *p* value = 0.00012) and vascular development (97 node genes; Adj *p* value = 0.00037). Ingenuity pathway analysis (Fig. 3) also revealed a predicted functional effect of altered miRNAs in pathways associated with placental development including cellular growth and proliferation, cellular movement, cell death and survival and cell-to-cell signalling and interaction.

### Sexually dimorphic maternal circulating miRNAs associated with FGR pathology

**Fig. 4:**
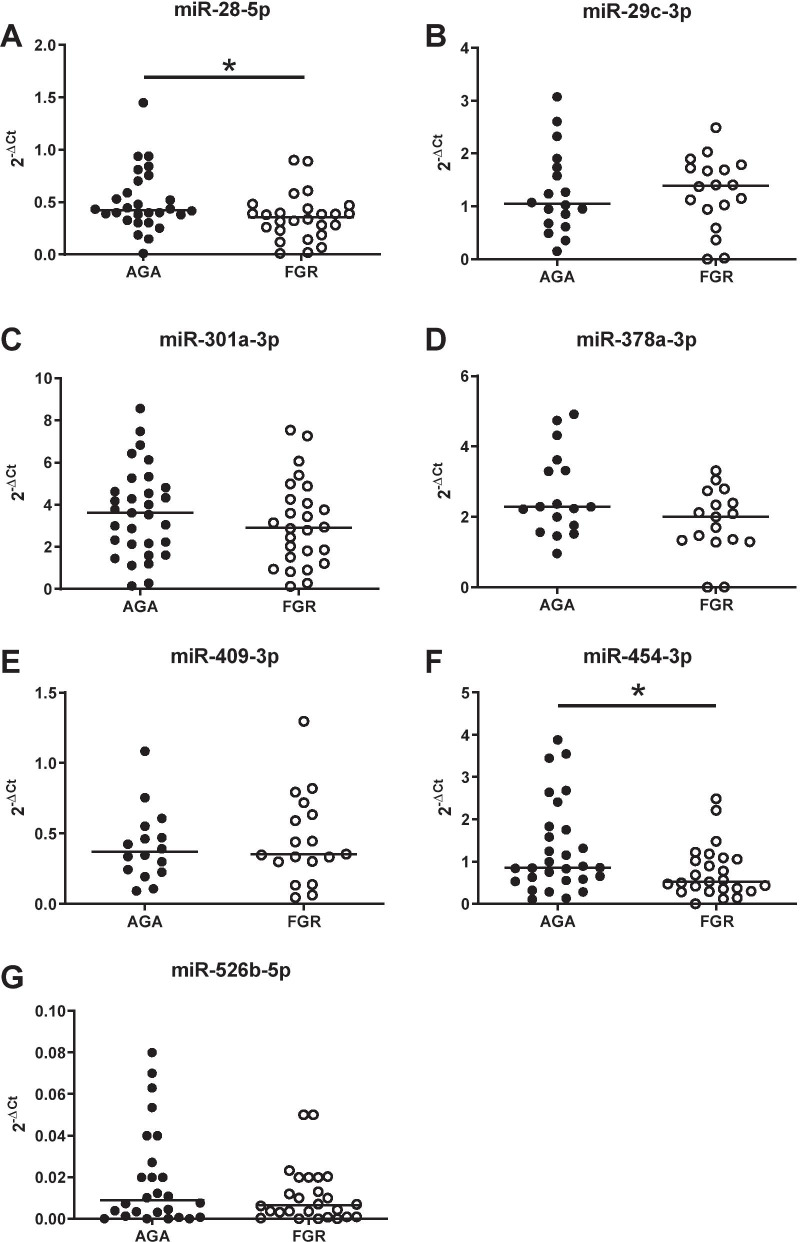
Q-PCR validation of candidate microRNAs in maternal serum in uncomplicated and FGR pregnancies. qPCR was performed on individual microRNAs isolated from maternal serum of women with appropriately grown (AGA; IBC 20–80th) or growth-restricted (FGR; IBC < 5th) infants using specific primers for **A** miR-28-5p, **B** miR-29c-3p, **C** miR-301a-3p, **D** miR-378a-3p, **E** miR-409-3p, **F** miR-454-3p and **G** miR-526b-5p. Data were normalised to 2 reference miRNAs and expressed as 2^−ΔCt^. Individual data points shown (*n* = 18–31/group); line represents the median. *p < 0.05 Mann–Whitney *U* test

**Fig. 5:**
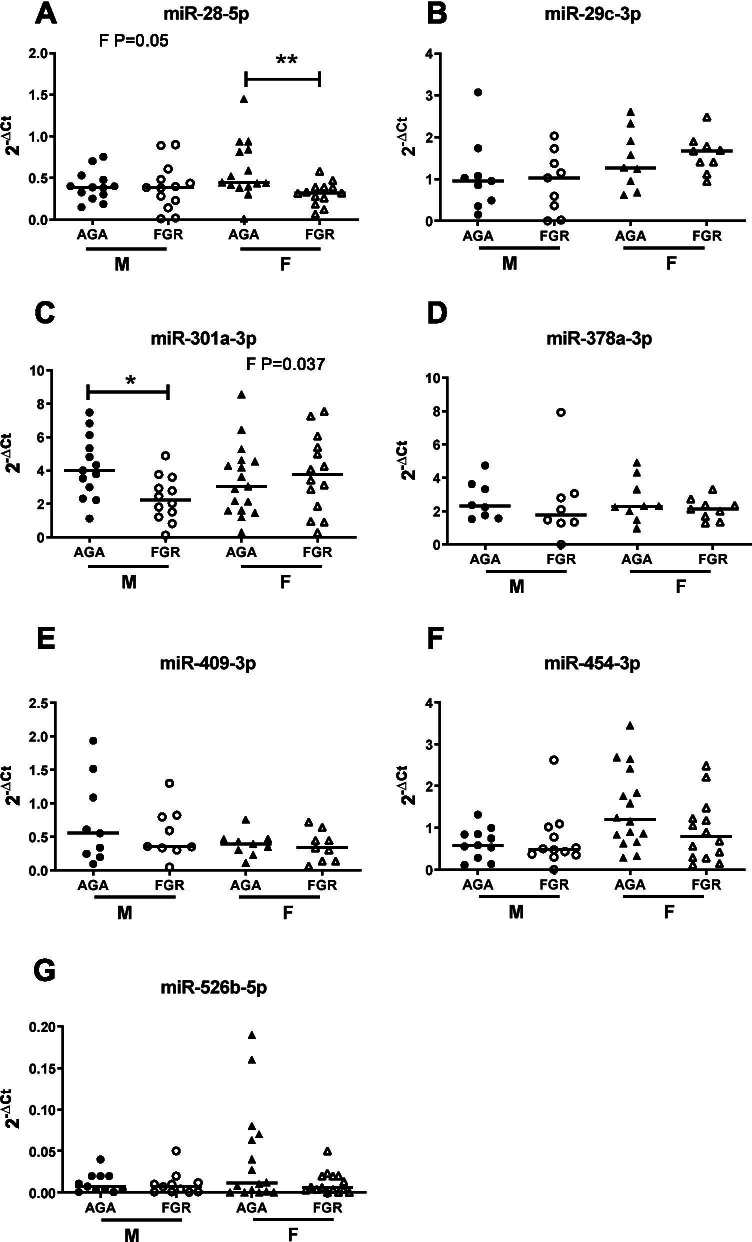
Effect of fetal sex on microRNA levels in maternal serum in uncomplicated and FGR pregnancies. qPCR was performed on individual microRNAs isolated from maternal serum of women with appropriately grown (AGA; IBC 20–80th) or growth-restricted (FGR; IBC < 5th) infants using specific primers for **A** miR-28-5p, **B** miR-29c-3p, **C** miR-301a-3p, **D** miR-378a-3p, **E** miR-409-3p, **F** miR-454-3p and **G** miR-526b-5p. Data were normalised to 2 reference miRNAs and expressed as 2^−ΔCt^. Data were stratified into male (*n* = 15/group) and female (*n* = 16/group). Individual data points shown, line represents the median. 2-way ANOVA with linear step-up multiple comparison test. Interaction (F) between the groups was considered significant when *p* < 0.05. Kruskal–Wallis followed by Dunn’s post hoc analysis was used to determine difference between individual groups; *p < 0.05, **p < 0.01

Subsequent analysis in a larger cohort of patients by QPCR revealed that only miRNA-28-5p and miR-454-3p levels were significantly reduced in FGR compared to normal pregnancies (*p* = 0.028 and *p* = 0.045; Fig. 4A and F, respectively). miR-378a showed a trend for lower levels in FGR pregnancies, but this did not reach significance (*p* = 0.08; Fig. 4D). Four remaining miRNAs (miR-29c-3p, miR-301a-3p, miR-409-3p and miR-526b-5p; Fig. 4B, C, E, G), were unaltered between groups. However, male fetuses have poorer outcomes and are at higher risk of FGR and stillbirth [29, 64] than their female counterparts [29, 64]. Whilst mechanisms remain unclear, sex-specific differences in miRNA expression between placentas of male and female fetuses have been reported [27, 30]. When initial miRNA array data were analysed by sex, rather than pathology, sex differences between four miRNAs: miR-29c-3p, miR-32-5p, miR-136-5p (all increased in male samples) and miR-520 h (increased in female), were observed (Table 4). Whilst only one of these miRNAs, miR-29c-3p, was found to also be altered in FGR miRNA array data (Table 3), it suggested that there was potentially sexual dimorphism in miRNA levels in FGR pregnancies. QPCR data obtained for the seven miRNAs assessed in the larger cohort of patient samples was stratified by fetal sex to determine if there were sexual dimorphic patterns for these serum miRNAs in FGR (Fig. 5). Using 2-way ANOVA miR-29c-3p (*p* = 0.031; Fig. 5B), miR-526b-5p (*p* = 0.0224; Fig. 5G) and miR-454-3p (*p* = 0.014; Fig. 5F) were found to have sexually dimorphic expression independent of fetal growth. Additionally, altered miR-301a-3p and miR-28-5p levels in FGR pregnancies were sex-specific; specifically miR-301a-3p was 45% lower in FGR pregnancies when the fetus was male (4.22 ± 1.87 vs 2.33 ± 1.35 Male AGA vs FGR (mean ± standard deviation); interaction (F) *p* = 0.037; Fig. 5C) but not female, and miR-28-5p was reduced by 28% in FGR pregnancies with a female fetus (0.59 ± 0.34 vs 0.32 ± 0.14 AGA vs FGR (mean ± standard deviation); interaction (F) *p* = 0.05; Fig. 5A), but not when the fetus was male. No other miRNAs assessed showed sexually dimorphic patterns.

**Table 4:** Differentially expressed microRNAs between female and male serum samples identified by microarray

miRNA ID	SD F	SD M	Average dCq F	Average dCq M	Fold-change	*p*-value	Sexual dimorphism	Reported association with pregnancy pathology
hsa-miR-520 h MIMAT0002867	0.40	0.66	− 2.1	− 3.5	2.6	0.018		↑ In plasma EVs in GDM pregnancies [19] ↑1st trimester plasma in women who develop PE [65] ↑ Placental expression in PTB [66] ↓ Placental expression in FGR [49]
hsa-miR-29c-3p MIMAT0000681	0.28	0.17	2.0	2.5	− 1.4	0.038	↑ M plasma [39] ↑ F cerebellum [40]	↑ Plasma levels in gestationally obese women [51]
hsa-miR-32-5p MIMAT0000090	0.37	0.15	1.2	1.8	− 1.5	0.039		↓Placental expression in PE [67]
hsa-miR-136-5p MIMAT0000448	0.69	0.6	− 2.5	− 1.4	− 2.2	0.046		↑ In plasma EVs in GDM pregnancies [19] ↑Expression in chorionic membranes in PTL [68]

When comparing the F group to the M group (combined FGR and AGA) using a *t*-test, 4 microRNAs were found to be differentially expressed using a cutoff of *p*-value < 0.05. (*n* = 4/group). Evidence of sexual dimorphism and/or association with pregnancy pathologies was determined by PubMed literature search accessed 30 May 2021. *SD* standard deviation, *F* female, *M* male, dCq = (Cq _target miRNA_ – Cq _reference miRNAs_), *EVs* extracellular vesicles, *GDM* gestational diabetes mellitus, *PE* pre-eclampsia, *PTB* pre-term birth, *FGR* fetal growth restriction, *PTL* pre-term labour

### Serum miRNAs correlate with hPL, a marker of placental dysfunction

**Fig. 6:**
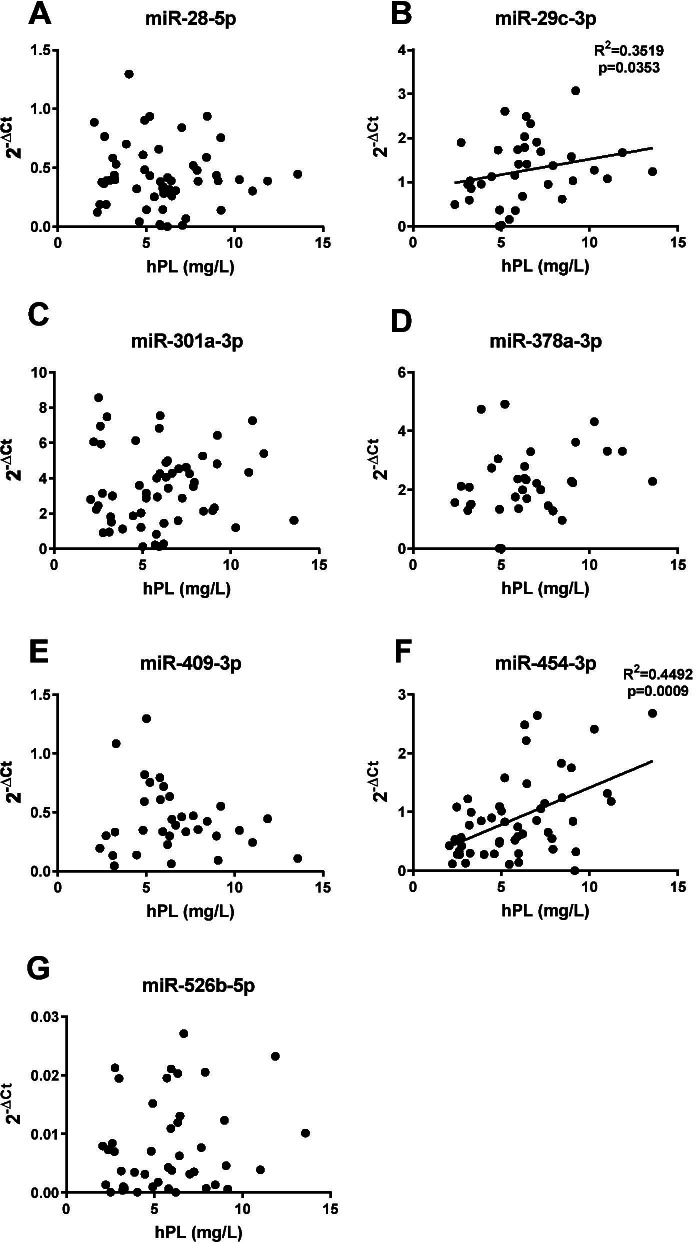
Relationship between circulating microRNAs and human placental lactogen (hPL) in maternal serum. Correlation between serum miRNA expression measured by qPCR and hPL a hormone marker of placental dysfunction detected by ELISA for **A** miR-28-5p, **B** miR-29c-3p, **C** miR-301a-3p, **D** miR-378a-3p, **E** miR-409-3p, **F** miR-454-3p and **G** miR-526b-5p. Positive correlations with hPL were detected for **B** miR-29c-3p (*r*^2^ 0.3519, *p* < 0.05) and **F** miR-454-3p (*r*^2^ 0.449, *p* < 0.001). Individual data points shown (*n* = 34–56), line represents the median. Spearman rank correlations

To determine whether levels of serum miRNAs altered in FGR could be indicative of placental dysfunction, the relationship between altered miRNA levels and maternal concentrations of hPL, an established marker of placental endocrine function in late pregnancy which is lower in pregnancies complicated by FGR [57, 58, 69], were assessed (Fig. 6). Positive correlations were found for miR-454-3p (*p* < 0.001, *r* = 0.449; Fig. 6F) and miR-29c-3p (*p* < 0.05, *r* = 0.352; Fig. 6B), but no associations were found for other miRNAs.

**Fig. 7:**
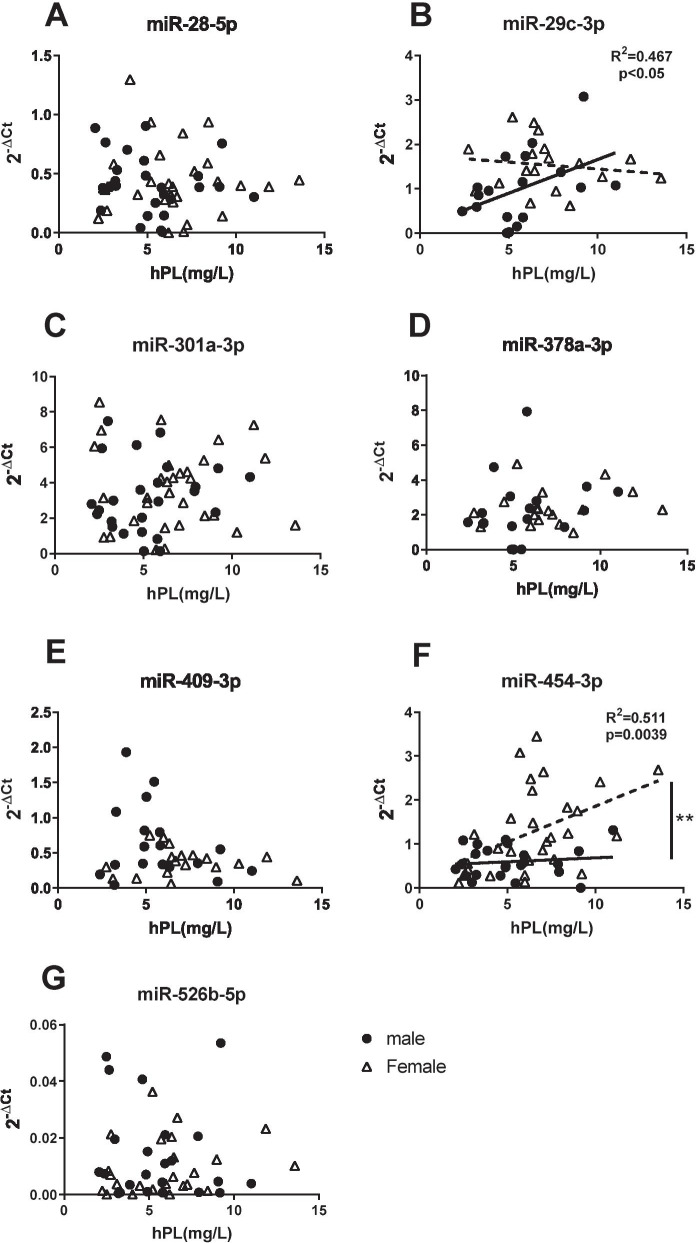
Effect of fetal sex on correlation between microRNAs and hPL in maternal serum. Correlation between maternal serum miRNA expression from male or female pregnancies measured by qPCR and hPL a hormone marker of placental dysfunction detected by ELISA for **A** miR-28-5p, **B** miR-29c-3p, **C** miR-301a-3p, **D** miR-378a-3p, **E** miR-409-3p, **F** miR-454-3p and **G** miR-526b-5p. Positive correlations with hPL were detected for **B** miR-29c-3p (*r*^2^ (*r* = 0.467, *p* =  < 0.05) in males only and **F** miR-454-3p (*r*^2^ *r* = 0.511, *p* < 0.010) in females only. Significant difference detected in intercept/elevation for **F** miR-454-3p (*p* < 0.01, linear regression). Individual data points shown (*n* = 17–27 males, *n* = 18–32 females/group), line represents the median. Spearman rank correlations

Further stratification of the data, based on fetal sex, revealed that the positive association between miR-454-3p and hPL, was only in pregnancies with a females fetus (*r* = 0.511, *p* < 0.01; Fig. 7F), whilst miRNA-29c-3p was only correlated with hPL when there was a male fetus (*r* = 0.467, *p* =  < 0.05; Fig. 7B). No sex-specific associations with hPL were detected for miRNAs 28-5p, 301a-3p, 378-3p, 409-3p or miR-526b-5p.

### Predicted functional roles of altered miRNAs

For a biomarker to be clinically useful, it must reliably be associated with the underlying pathology of a disease [70]. Circulating miRNAs have recently been reported to enter tissues where they interact with target mRNA and affect cellular function [71], thus, in addition to having potential biomarker utility, altered circulating miRNAs may also be causative for underlying pathologies. To assess whether the maternal serum miRNAs have the potential to contribute to placental dysfunction associated with FGR, in silico analysis was performed to determine experimentally validated downstream target genes of the sexually dimorphic miRNAs that were altered in maternal serum and/or correlating with hPL in FGR pregnancies.

Experimentally validated downstream targets for these miRNAs included nutrient transporters, cell cycle or apoptosis regulators and growth/angiogenic factors (Table 5). Many of these are altered in FGR pregnancies, e.g. vascular endothelial growth factor A (VEGFA) [72], pregnancy-associated plasma protein A (PAPPA) [73], insulin-like growth factor 1 (IGF-I) [74], insulin-like growth factor 2 receptor (IGF2R) [75], large neutral amino acid transporter (LAT1) [76], apoptosis regulators Bcl-2-like protein 11 (BCL2L11), tumour protein p53 (TP53), and B-cell lymphoma 2(BCL2) [77, 78] and the taurine transporter SLC6A6 [79].

**Table 5:** Bioinformatic analyses of miRNAs altered in maternal serum or correlating with hPL in FGR pregnancies

miRNA ID	FGR altered miRNA (QPCR data)	Sexual dimorphism (Current dataset)	Correlation with biochemical marker of placental dysfunction (hPL)	Selected gene targets Evidence level: -**Strong** (bold) -Weak (unbolded) -*Predicted* (italics)
miR-28-5p MIMAT0000085	Yes	↓in FGR females (QPCR)	No	**CDKN1A, MAPK1, IGFI, RAP1B, MAD2L1,** SLC7A5, CCND3 *PAPPA, CASP3*
miR-29c-3p MIMAT0000681	No	↑ males in microarray + ve correlation [hPL] males	Yes *R*^2^ = 0.467 *p* < 0.05	**CDK6, BCL2, MMP2, PTEN, AKT3, LAMC1, LAMC2, DNMT3A, COL1A, COL3A, FBA, FBB, FBG, PDGFRB** *VEGFA*
miR-301a-3p MIMAT0000688	Yes	↓ in FGR males (QPCR)	No	**BCL2L11, PAI-1, RUNX3, PTEN, NKRF, TGFβR2, DNMT1,** ESR1, XIAP, IGF2R, *PUMA, SLC6A6, IGF1, PPARG*
miR-454-3p MIMAT0003885	Yes	+ ve correlation [hPL] females	Yes *R*^2^ = 0.511 *p* < 0.01	**SMAD4, TGFβR2, ALK7**, **CXCL12,** TP53, IGF2R, CCND2, MAPK1, XIAP, PARP1, SLC38A2, *SLC6A6, IGF1, PPARG, DICER1, H19*

Potentially relevant gene targets selected from literature-based databases. Bold** = **experimentally validated as a target using STRONG methods (reporter assay, Western blot, qPCR); unbold = experimentally validated as a target using WEAK methods (microarray, NGS, pSILAC, other); italics = predicted target but not experimentally validated. *FGR* fetal growth restriction, *hPL* human placental lactogen. Correlations analysed using Spearman test

## Discussion

The current study identified 11 miRNAs that were altered in maternal serum of women that gave birth to a severely FGR infant compared to those delivering AGA babies. In silico analysis of the altered miRNAs revealed that all were present in a gene regulatory network with known roles in placenta dysfunction associated with FGR. Assessment of the altered miRNAs in a larger cohort of patients revealed that the association between levels of some maternal circulating miRNAs and FGR was dependent on fetal sex. Similarly, the association between maternal circulating miRNAs and hPL was fetal sex-dependent.

**Fig. 8:**
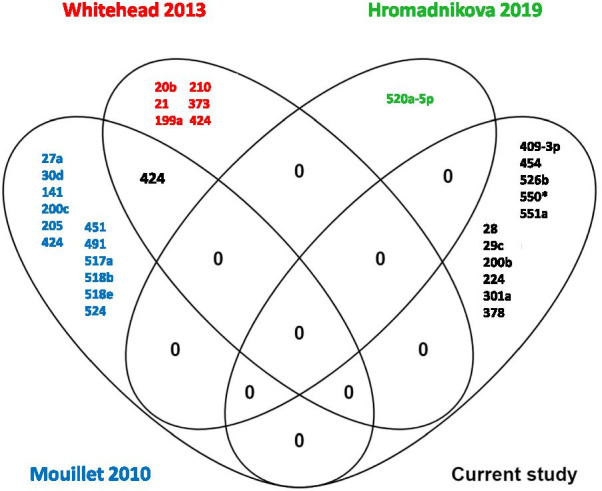
Venn diagram showing the overlapping microRNAs identified from studies comparing maternal serum from uncomplicated and FGR pregnancies. Current study compared with Mouillet et al. [22], Whitehead 2013 [23] and Hromadnikova 2019 [24]

To date, there have been limited studies of maternal serum miRNA in pregnancies complicated by FGR and existing data are not in agreement [22–25, 49]. In our current investigation, there was no overlap with any of the miRNAs detected in the previous studies even though they were all present in the array used (Fig. 8). However, our approach differed from prior studies in several ways including: methodology [22, 24], classification of FGR with our study focused on later onset FGR and examining the influence of fetal sex. We initially focussed on later onset FGR because the majority of cases of FGR are late-onset and our current ability to detect FGR and fetal compromise is poorer [6, 23, 25, 80]. Although our study was designed to address potential limitations of previous studies, restricting the gestational age window of samples in the initial screen may have excluded some miRNAs seen in earlier studies. Differences between samples and study designs likely account for the lack of overlapping miRNAs found in any published studies.

Sex-specific differences in miRNA expression between placentas of male and female fetuses may also contribute to the observed differences between published studies. We found sex differences in miR-526b, miR-454-3p and miR-29c-3p in maternal serum, independent of fetal growth. miR-454-3p and miR-29c-3p are also expressed in a sex-dependent manner in other tissues [39, 40, 54] so accounting for fetal sex when considering the contribution of these miRNAs to disease processes is of obvious importance.

Here, we also report that two miRNAs, miR-28-5p and miR-301a-3p are altered in FGR pregnancies only when there is a female or male baby, respectively. Whilst there are no reports of sex-differences in miR-28-5p during pregnancy, the reduction of miR-301a-3p only in FGR pregnancies with a male fetus, is consistent with previous reports of sexual dimorphic changes in miR-301a-3p in maternal serum in pre-eclampsia (PE) [43]. It is possible therefore, that miR-301a-3p and miR-28-5p could be used to predict FGR specifically in pregnancies with male or female babies. Caution should be taken however, since the heterogeneity of FGR pathophysiology means these miRNAs may only be useful in predicting FGR in the subpopulation of FGR cases in which they were originally identified, that is where the infant is < 3rd centile at birth. Prospective studies in a large independent cohort are required to assess this.

In addition to the potential biomarker utility of these miRNAs, in silico analyses reveal potential functional roles for the altered miRNAs. These miRNAs are enriched in functional networks associated with cellular response to stress, cell proliferation and vascular development. Since these are all hallmarks of placental dysfunction in FGR pregnancies [13, 81, 82], it is possible that they may contribute to the aetiology of FGR by impacting on placental function. Indeed placental sexual dimorphism is well documented [28, 30, 45, 83, 84] with male fetuses having increased susceptibility to adverse perinatal outcomes [64, 85, 86]. It would therefore be interesting to assess whether the sexually dimorphic profiles observed for serum miRNAs are also reflected in placental tissue, and whether the miRNAs have functional roles in the placenta. Indeed, the relationship between some of the altered miRNAs and hPL—a marker of placental dysfunction together with a functional study showing that overexpression of miR-454-3p in trophoblast increases proliferation and invasion whilst reducing apoptosis [55], would support this hypothesis. Roles for the altered miRNAs in other tissues combined with information on their validated targets also provides further support for roles of these miRNAs in regulation of placental function.

IGF-I, a critical regulator of normal human placental development [87, 88], is a validated gene target of miR-28-5p [89] and is strongly associated with FGR in both animal models [90] and human pregnancies [91]. In a study of healthy pregnancies, IGF-I concentrations in cord blood were found to be higher in women carrying a female infant [92]. Target genes of miR-29c-3p and miR-301a-3p in cell death and survival pathways [93–95] may be relevant to FGR as these are known to be altered in FGR pregnancies [77, 96, 97], however further studies are required to assess this.

### Perspectives and significance

This manuscript contributes to an increasing understanding of miRNAs in the maternal circulation and how these may be used to predict differing susceptibility of male and female fetuses to FGR and stillbirth. These studies have identified sexually dimorphic miRNA profiles in maternal circulation in pregnancies, specifically those that result in FGR infants. Further studies to establish where these miRNAs originate and investigate potential roles for these miRNAs in the placenta may provide greater understanding of the pathogenesis of placental dysfunction. In addition, it is possible that sex-specific altered circulating miRNAs could contribute to the differences in frequency of pregnancy complications such as increased incidence of term pre-eclampsia and GDM and stillbirth in women carrying a male fetus [86, 98]. At present, fetal sex is not considered in the management of pregnancy conditions, thus the management of cases included here and the observation of increased frequency of complications will not have altered depending upon fetal sex.

Identifying novel predictive biomarkers that can be used in conjunction with existing techniques is important for better predicting late-onset FGR and stillbirth and reducing associated morbidity and mortality [99]. Existing algorithms to detect late-onset placental dysfunction (UAD impedance and PlGF/sFlt1 ratio in maternal serum) [100] may be strengthened by inclusion of one or more of these miRNAs or combining miRNAs with recently identified circulating mRNA markers such as EMP1, which is increased in women who subsequently suffered a stillbirth [101]. Further studies on an extended sample set are necessary to test this hypothesis. Including fetal sex in these algorithms (which would be necessary to interpret the miRNA data) may be challenging as fetal sex is not always accurately determined on ultrasound, parents may not wish to know the sex of their baby and in some states (e.g. India), antenatal determination of fetal sex is prohibited. Nevertheless, the clinical utility of combinations of biomarkers (including fetal sex) should be explored, and if deemed effective in reducing fetal mortality and morbidity, fetal sex could be included.

## Conclusions

Our study has identified a distinct profile of circulating miRNAs in women with FGR infants < 3rd centile. Two of these miR-28-5p and miR-454-3p, were also altered in a larger population of pregnancies with infants < 5th centile. Further analyses identified sexually dimorphic changes in some of the altered miRNAs; both miR-28-5p and miR-301a-3p to be altered in maternal serum in FGR pregnancies according to infant sex and miR-29c-3p and miR-454-3p showed sexually dimorphic relationship to placental dysfunction. Furthermore, these altered miRNAs are linked to placental regulatory gene networks. Further investigations to determine the source of these miRNAs and their relationship to placental dysfunction will lead to a better understanding of the relationship between circulating miRNAs and placental dysfunction in FGR and may enable the development of future treatments for placental dysfunction. Ultimately, further studies are required to determine the potential for these altered miRNAs to provide potential biomarkers to predict differing susceptibility of male and female fetuses to FGR and stillbirth.

## Supplementary Information

**Additional file 1.** List of miRNAs in Exiqon QPCR array Human panels I + II.

**Additional file 2: Table S1.** Target sequences of LNA microRNA primer sets utilised for microRNA Q-PCR.

## Data Availability

The microarray data generated during the current study has been deposited in NCBI's Gene Expression Omnibus and is accessible through GEO Series accession number GSE188186 (https://www.ncbi.nlm.nih.gov/geo/query/acc.cgi?acc=GSE188186).
