# Supplementary material for: Sexually dimorphic patterns in maternal circulating microRNAs in pregnancies complicated by fetal growth restriction
Source: Biol Sex Differ. 2021 Nov 17;12:61. doi: 10.1186/s13293-021-00405-z (PMC8597318; doi:10.1186/s13293-021-00405-z)
Supplement: Supplementary file 2 — Additional file 2: Table S1. Target sequences of LNA microRNA primer sets utilised for microRNA Q-PCR. [file 13293_2021_405_MOESM2_ESM.docx]

| **miRNA ID** | **Primer target sequence**  **(5’-3’)** | **Accession number** |
| --- | --- | --- |
| **hsa-miR-23a-3p** | AUCACAUUGCCAGGGAUUUCC | MIMAT0000078 |
| **hsa-miR-28-5p** | AAGGAGCUCACAGUCUAUUGAG | MIMAT0000085 |
| **hsa-miR-29c-3p** | UAGCACCAUUUGAAAUCGGUUA | MIMAT0000681 |
| **hsa-miR-191-5p** | CAACGGAAUCCCAAAAGCAGCUG | MIMAT0000440 |
| **hsa-miR-301a-3p** | CAGUGCAAUAGUAUUGUCAAAGC | MIMAT0000688 |
| **hsa-miR-378a-3p** | ACUGGACUUGGAGUCAGAAGGC | MIMAT0000732 |
| **hsa-miR-409-3p** | GAAUGUUGCUCGGUGAACCCCU | MIMAT0001639 |
| **hsa-miR-451a** | AAACCGUUACCAUUACUGAGUU | MIMAT0001631 |
| **hsa-miR-454-3p** | UAGUGCAAUAUUGCUUAUAGGGU | MIMAT0003885 |
| **hsa-miR-526b-5p** | CUCUUGAGGGAAGCACUUUCUGU | MIMAT0002835 |

**Table S1. Target sequences of LNA microRNA primer sets utilised for microRNA Q-PCR.**
